# Biosensing Near the Exceptional Point Based on Resonant Optical Tunneling Effect

**DOI:** 10.3390/mi12040426

**Published:** 2021-04-14

**Authors:** Yang Liu, Pengyun Yan, Feng Liu, Aoqun Jian, Shengbo Sang

**Affiliations:** 1 MicroNano System Research Center, Taiyuan University of Technology, Taiyuan 030024, China; liuyang0294@link.tyut.edu.cn (Y.L.); yanpengyun0255@link.tyut.edu.cn (P.Y.); yangting0282@link.tyut.cn (F.L.); 2 Key Laboratory of Advanced Transducers and Intelligent Control System, Shanxi Province and Ministry of Education, Taiyuan 030024, China

**Keywords:** exceptional point, biosensing, resonant optical tunneling effect, carcinoembryonic antigen

## Abstract

Inspired by exceptional point (EP) sensing in non-Hermitian systems, in this work, a label-free biosensor for detecting low-concentration analytes is proposed, via a special multilayer structure: a resonant optical tunneling resonator. Due to the square root topology near the exceptional point, a recognized target analyte perturbs the system deviated from the exceptional point, leading to resolvable modes splitting in the transmission spectrum. The performance of the designed sensor is analyzed by the coupled-mode theory and transfer matrix method, separately. Here, the simulation results demonstrate that the obtained sensitivity is 17,120 nm/imaginary part unit of refractive index (IP) and the theoretical detection limit is 4.2 × 10^−8^ IP (regarding carcinoembryonic antigen (CEA), the minimum detection value is 1.78 ng). Instead of the typical diffusion manner, the liquid sample is loaded by convection, which can considerably improve the efficiency of sample capture and shorten the response time of the sensor. The sketched sensor may find potential application in the fields of biomedical detection, environment protection, and drinking water safety.

## 1. Introduction

In quantum mechanics, the exceptional point (EP) was first proposed in the perturbation of linear non-Hermitian operators [1], at which the eigenvalues and the corresponding eigenstates will be degenerated [2]. Due to gain and loss being easily integrated and adjusted in optical materials, EPs have been extensively realized in optics [3,4,5,6,7]. Several optical systems, such as microcavities [8], optical waveguide [9], and nonlinear photonic crystal [10] have been widely investigated to explore the characteristics of EPs, and series of the counterintuitive phenomena have been observed in these optical systems at EPs [11,12,13]. Among them, a striking feature of EPs is their unique response of degenerating eigenvalues to the weak perturbation. When the external perturbation is induced in the system, the formerly degenerated eigenvalues are split. For an Nth-order EP at which N eigenvalues coalesce [1], the modes splitting is proportional to the Nth root of perturbation, which is quite different from the classic linear response. Since Wiersig revealed the general principle of the ultra-low-resolution sensing based on such modes splitting and adopted this unique feature for the ultra-low-resolution sensing [14,15], the nanoparticle detection sensor was later verified in experiment [16]. Afterward, sensitivity has been enhanced 23 times at three-order EPs for thermal sensing [17].

As a special optical phenomenon, the resonant optical tunneling effect (ROTE) was discovered and later verified in experiments successively [18,19]. The ROTE resonators provide the benefits of low cost, effortless equipping, easy fabrication and integration. Several ROTE-based sensors, such as acceleration sensors [20] and refractive index (RI) sensors [21,22], have been theoretically or experimentally demonstrated. An EP biosensor was proposed and designed by a parity-time (PT) coupled symmetric ROTE resonator [23] for low-concentration carcinoembryonic antigen (CEA) detection. As one of the top 10 discoveries in physics [24], although PT symmetry is a special case of non-Hermitian systems used to study its characteristics, the operation of the sensor based on EPs in a PT symmetric system suffers from unstable mode detuning. General non-Hermitian systems have superiority in avoiding experimental complexity and instability [25], providing a new opportunity to explore the applications of EPs. Given the above research background, considering the complexity of balancing the gain and loss of two coupling cavities required by a PT symmetric system structure in experiments, a simply constructed structure with two directly coupled loss cavities was further theoretically studied. Not the PT symmetrical structure, but the more general non-Hermitian system was selected as the physical model for the sensor development.

In this study, a biosensor based on the mode splitting near the EP in the coupled ROTE resonators system was constructed for CEA detection. We designed a ROTE seven-layer structure, and its sensing principle was discussed here. The transmission spectra and the evolution of eigenvalues were simulated in a mathematical model. Finally, the performance of the designed sensor was theoretically evaluated, finding the sensitivity is up to 17,120 nm/IP. The designed biosensor has application potential in the fields of cancer diagnosis, drug screen, drinking water safety, and biomedical discoveries.

## 2. Device Design

**Figure 1:**
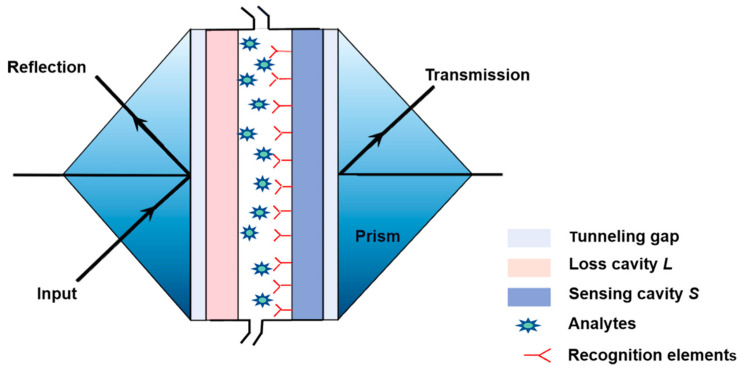
Schematic graph of the biosensor structure based on the resonant optical tunneling effect (ROTE) structure.

A schematic of the designed biosensor structure based on the coupled ROTE resonators is shown in Figure 1. The materials and parameters of the device are listed in Table 1. In experiments, the preparation processes of the designed device are as follows: Two silicate layers are used as the coupled cavities (the loss cavity *L* and the sensing cavity *S*). The polymer layers (low RI layers) are formed on the silicate glass wafer by spin coating as the tunneling layer of the ROTE structure. Then, the glass prism and the tunneling layer are assembled using ultraviolet curing adhesive, and two prisms are placed on the micro-positioners to approach each other. The behavior in the vicinity of the EP is controlled via adjusting the tiny separation between the two resonators (coupling strength *K*). The surface of the sensing cavity *S* is biofunctionalized in advance to bind specific target analytes, which are checked on the sensing surface perturbing the system from its EP, leading to modes splitting. Finally, the gap between two resonator units is sealed by the ultraviolet curing adhesive to form a closed sample-loading channel. Compared with the whispering-gallery-mode structure, the ROTE structure can be fabricated by a simple process and is easy to integrate. Additionally, the coupling layer can be used as a fluid channel in this special sensor structure, so that the samples are loaded by convection rather than diffusion, which can greatly improve the capture efficiency of analytes and shorten the response time of the sensor [26]. As diffusion relies only on the mass transfer of molecular motion, it expends on the detection time and thus reduces capture efficiency [27]. On the other hand, the convection is the mass transfer caused by fluid motion through the designed microchannel; and the analytes interact with the sensing surface almost instantaneously.

CEA was selected as the target analytes for our designed sensor. As a tumor marker, the concentration of CEA in tumor tissue is significantly higher than in normal tissue [28]. The detection of CEA concentration in exhaled breath condensate (EBC), as a noninvasive and simple detection method, can be used for the early diagnosis of lung cancer [29]. CEA manifests high specificity in patients with adenocarcinoma [30]. Generally, the concentration of CEA in EBC is about 2 ng/mL for healthy people and above 4 ng/mL for patients [31]. CEA can be specifically captured by the antigen-antibody reaction. Therefore, the surface of the sensing cavity *S* needs to be biofunctionalized to recognize CEA. This specific antigen and antibody recognition induces the absorption on the cavity *S*, which can be analyzed by the degree of splitting of the modes according to the evolution of the spectral line at the EP.

## 3. Theoretical Analysis and Simulation

**Figure 2:**
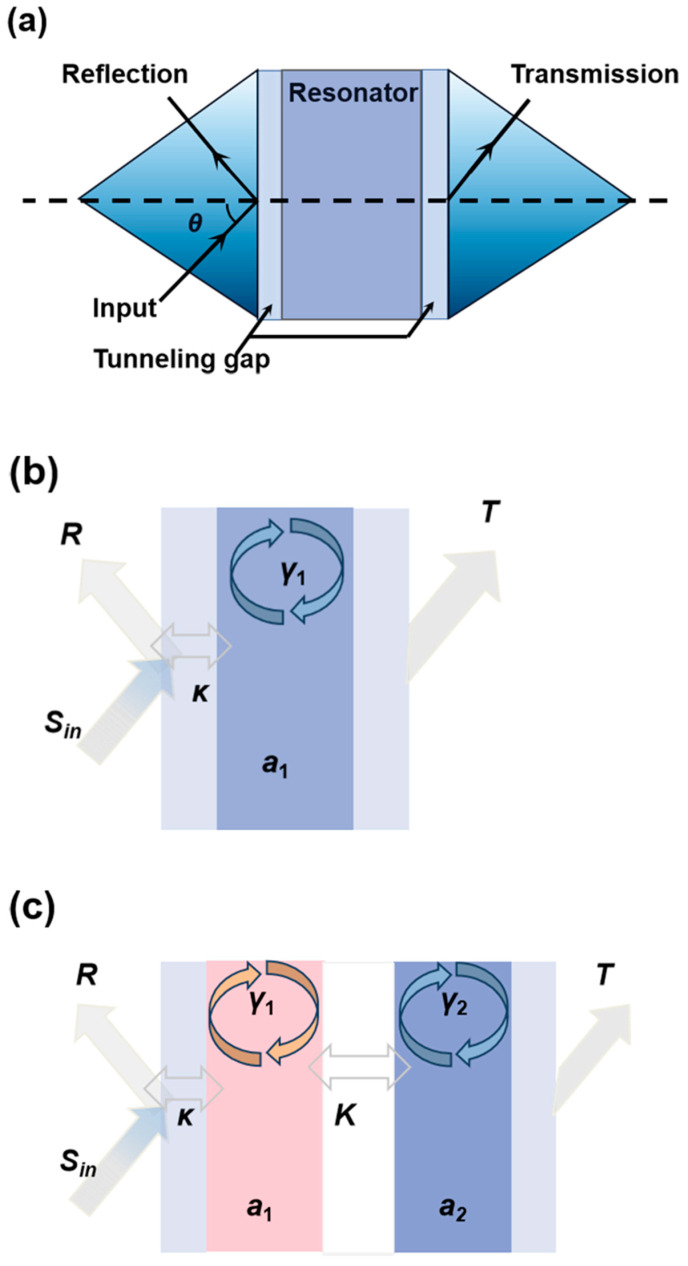
Schematic diagram of the ROTE resonator (**a**), the ROTE resonator model (**b**), and the coupled ROTE resonators model (**c**).

Originating from the optical tunneling effect (frustrated total internal reflection, FTIR), the ROTE refers to the resonance effect of the tunneled light in a well-designed resonator, as seen in Figure 2. The typical ROTE structure consists of five layers (input layer, first tunneling gap, central slab, second tunneling gap, and output layer) with an alternative RI distribution of high and low. When the incident angle is greater than the total reflection angle and the tunneling gap is thin enough, the incident light can pass through the tunneling gap and resonate in the central slab [21]. To analyze the manner of the EP, the coupled ROTE resonators model is designed as shown in Figure 2c. The coupling strength between the directly coupled resonators can be tuned by adjusting the width of the coupling layer.

To clearly illustrate the physical mechanism between the two resonator modes in this system, the dynamic equations of two directly coupled modes can be written as follows according to coupled-mode theory [25,32]:

(1) $$\{\begin{array}{l} \frac{da_{1}}{dt}=(i\Delta \omega_{1}-\gamma_{t})a_{1}+iKa_{2}+kS_{in}\\ \frac{da_{2}}{dt}=(i\Delta \omega_{2}-\gamma_{2})a_{2}+iKa_{1} \end{array},$$

where $a_{1}$ and $a_{2}$ denote the mode field amplitudes of two cavities; $\Delta \omega_{1,2}=\omega -\omega_{1,2}$, where ω is the frequency of the probe light, $\omega_{1,2}$ is the resonance frequencies of the resonators; $\gamma_{t}=\gamma_{1}+\gamma_{e}$, where $\gamma_{1}$ and $\gamma_{e}$ correspond to the intrinsic loss of the cavity *L* and the loss and the external loss induced by the excitation source, respectively; $\gamma_{2}$ is the intrinsic loss of the cavity *S*; *K* indicates the coupling strength between the two modes; *k* is the coupling strength among the cavity *L* and the excitation source; and $S_{in}$ represents the input field amplitude.

Solving Equation (1) at the steady state, the following expression is obtained:

(2) $$\{\begin{array}{c} a_{1}=-\frac{k(i\Delta \omega_{2}-\gamma_{2})S_{in}}{(i\Delta \omega_{1}-\gamma_{t})(i\Delta \omega_{2}-\gamma_{2})+{Κ}_{}^{2}}\\ a_{2}=i\frac{KkS_{in}}{(i\Delta \omega_{1}-\gamma_{t})(i\Delta \omega_{2}-\gamma_{2})+{Κ}_{}^{2}} \end{array}.$$

According to the relationship between the input and reflection port $r=S_{in}-ka_{1}$, the reflectance R can be given as:

(3) $$R={\left|1-k\frac{a_{1}}{S_{in}}\right|}^{2}={\left|1-\frac{k^{2}(\gamma_{2}-i\Delta \omega_{2})}{(i\Delta \omega_{1}-\gamma_{t})(i\Delta \omega_{2}-\gamma_{2})+{Κ}_{}^{2}}\right|}^{2}.$$

The eigenfrequencies of the supermodes of the coupled cavities are described by the non-Hermitian Hamiltonian $\overset{\Lambda }{H}=\left[\begin{array}{cc} \omega_{1}-i\gamma_{t} & K\\ K & \omega_{2}-i\gamma_{2} \end{array}\right]$ as:

(4) $$\omega_{\pm }=\frac{\omega_{1}+\omega_{2}}{2}-i\frac{\gamma_{t}+\gamma_{2}}{2}\pm \sqrt{{Κ}^{2}+{\left(\frac{\omega_{1}-\omega_{2}}{2}-i\frac{\gamma_{t}-\gamma_{2}}{2}\right)}^{2}}.$$

Assuming $\omega_{1}=\omega_{2}=\omega_{0}$, the simplified form of Equation (4) is:

(5) $$\omega_{\pm }=\omega_{0}-i\frac{\gamma_{t}+\gamma_{2}}{2}\pm \sqrt{{Κ}^{2}-\frac{{\left(\gamma_{2}-\gamma_{t}\right)}^{2}}{4}}.$$

Equation (4) can be further simplified as (defining $\Delta \omega_{\pm }$ as the difference between $\omega_{\pm }$ and $\omega_{0}$):

(6) $$\Delta \omega_{\pm }=-i\frac{\gamma_{t}+\gamma_{2}}{2}\pm \sqrt{{Κ}^{2}-\frac{{\left(\gamma_{2}-\gamma_{t}\right)}^{2}}{4}},$$

which are complex with a real part and an imaginary part. As expressed in Equation (6), the values of the two eigenfrequencies are determined by the square root term. $\eta =\left|\frac{\gamma_{2}-\gamma_{t}}{2}\right|$ quantifies the loss contrast of the resonators.

**Figure 3:**
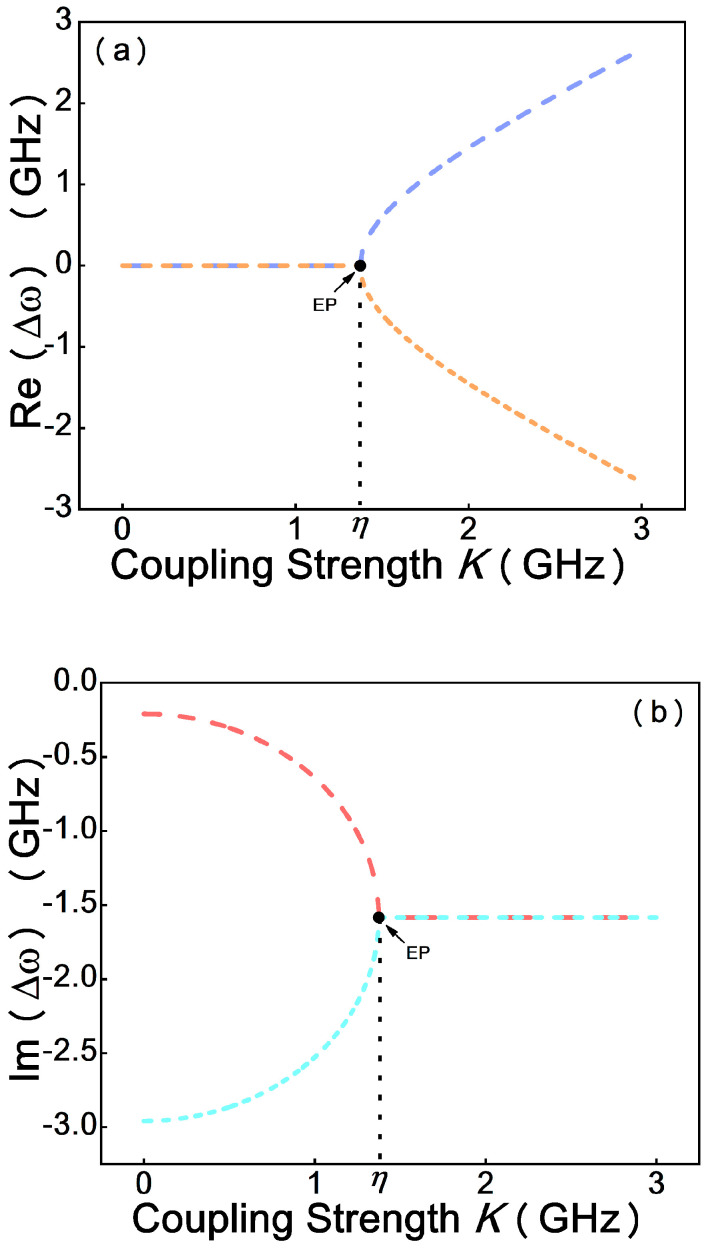
Evolution of the real (Re(Δω), (**a**)) and imaginary (Im(Δω) (**b**)) parts of the eigenvalues as the coupling strength increases.

Figure 3 shows the evolution of the eigenfrequencies with the relative ratio of coupling strength K and the loss contrast η, as described by Equation (6). By adjusting the coupling strength, the system can be operated at EP where the imaginary parts and real parts of the eigenvalues coalesce. In the case of low coupling strength, |K|<η, which quantifies the system, is in the weak coupling regime, the two eigenfrequencies have different imaginary parts but same real parts. Until the coupling strength increases to be |K|=η, the square root term is zero; that is, the degeneracy of the two eigenfrequencies is realized. When the coupling strength grows continuously, the system runs in the strong coupling regime, quantified by |K|>η; the two eigenfrequencies have different real parts but same imaginary parts. Therefore, the EP in the transition from the strong coupling regime to the weak coupling regime can be observed based on the simulation results.

**Figure 4:**
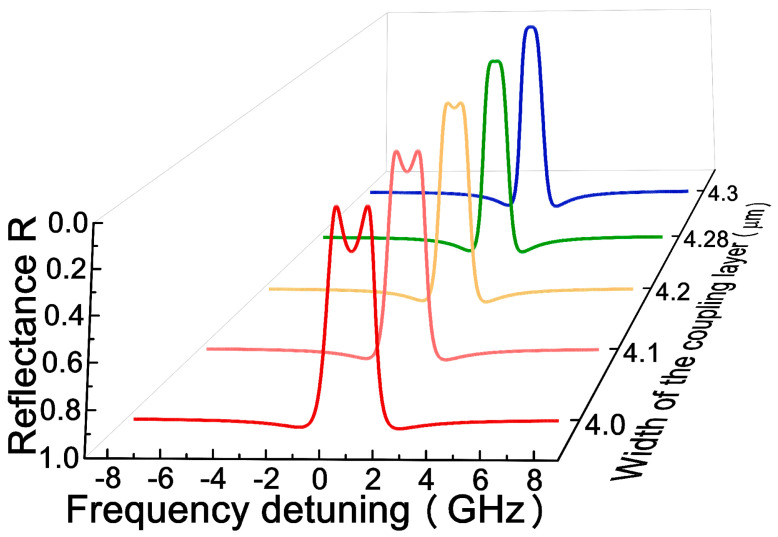
The transition of the output spectrum with the increase of the width of the coupling layer.

To further verify the results deduced by the coupled-mode theory, the transfer matrix method was used to calculate the output spectrum of the coupled ROTE resonators model as a multi-layer structure. Theoretically, the real and imaginary parts of the eigenvalues relate to the resonance frequencies and linewidths of the supermodes, respectively. The value of the coupling strength is opposite to the width of the coupling layer. Therefore, the transformation of the output spectrum reveals the effect of the coupling strength on the supermodes. As shown in Figure 4, when the width of the coupling layer is narrow, |K|>η, the supermodes have two different resonance frequencies, but the same linewidths, which results in two dips in the output spectrum. The spectral distance is quantified by δ ($\delta =2\sqrt{{Κ}^{2}-\frac{{\left(\gamma_{2}-\gamma_{t}\right)}^{2}}{4}}$). With the increase in layer width, the coupling strength decreases; if the coupling strength K is equal to η and the supermodes have same resonance frequencies, the spectrum shifts from double dips to a single dip. We find that by adjusting the width of the coupling layer, the EP can be realized for the coupled ROTE resonators model. Consequently, the evolution of the eigenvalues can be observed by tuning the coupling strength, and the state of system can be monitored by checking the transmission spectrum.

Introducing an additional loss into the cavity *S*, Equation (7) is obtained:

(7) $$\Delta \omega_{\pm }=-i\frac{\gamma_{t}+\gamma_{2}+\gamma_{a}}{2}\pm \sqrt{{Κ}^{2}-\frac{{\left(-\gamma_{t}+\gamma_{2}+\gamma_{a}\right)}^{2}}{4}}.$$

**Figure 5:**
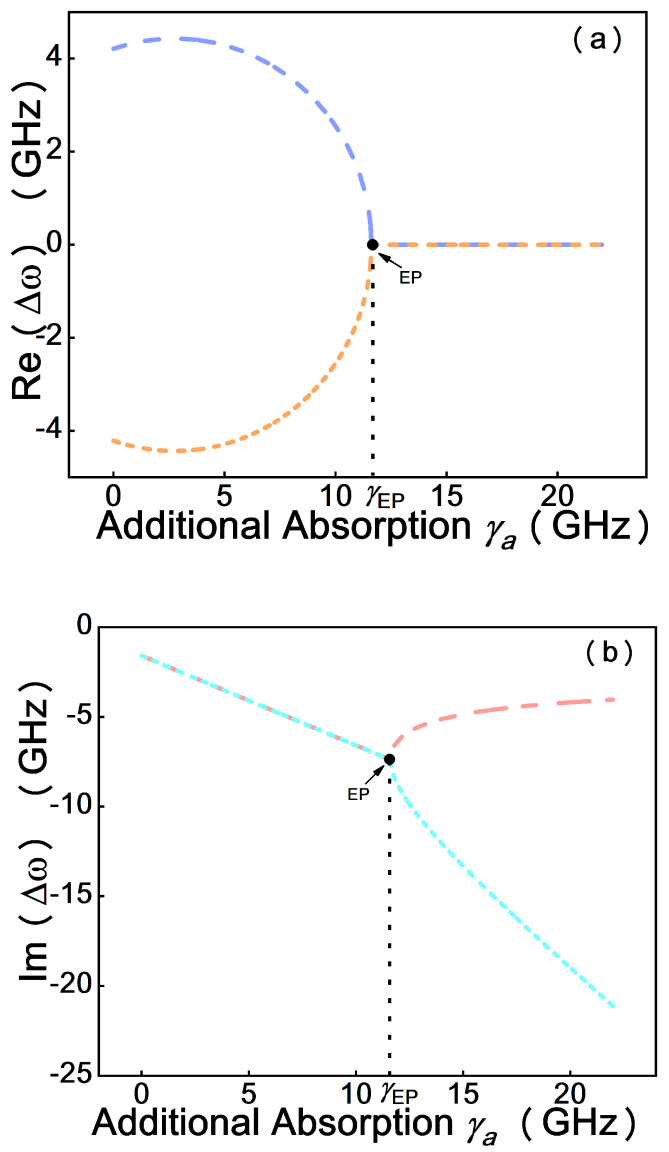
The real (Re(Δω), (**a**)) and imaginary (Im(Δω), (**b**)) parts of the eigenvalues vary with the increase in additional absorption of the sensing cavity.

The eigenvalues will be altered with the varying extra absorption $\gamma_{a}^{}$. The $\gamma_{EP}^{}$ value ($\gamma_{EP}=(\gamma_{t}-\gamma_{2})\mp 2k_{0}$) of the system at the EP is marked where the eigenvalues coalesce. The transformation of the complex eigenvalues as a function of $\gamma_{a}^{}$ is shown in Figure 5. When $\gamma_{\alpha }<\gamma_{EP}$, the two eigenfrequencies have different real parts, the imaginary parts are equal but not zero. At $\gamma_{\alpha }=\gamma_{EP}$, the eigenfrequencies degenerate to an identical pure imaginary number. As a result, after passing a critical value of the additional loss ($\gamma_{\alpha }>\gamma_{EP}$), the imaginary part of one supermode becomes more lossy, whereas the other mode has the opposite trend. In short, the EP can be observed in this system by altering the coupling strength and loss parameters alone.

The spectral translation near the EP was used to analyze the external absorption for CEA concentration detection in this designed sensor. To further evaluate the performance of the sensor, the effect of the absorption on the supermodes near the EP was discussed theoretically. In the simulation, the absorption of the cavities (*L* and *S*) is reflected by the imaginary part (IP) of the corresponding RI layers. The system runs near the EP by adjusting the coupling strength. In this situation, |K|=η. Therefore, the function of eigenfrequencies splitting with additional absorption can be described as $\Delta \omega_{diff}=2\sqrt{\frac{\varepsilon (\gamma_{t}-\gamma_{2})}{2}-\frac{\varepsilon^{2}}{4}}$. The equation reveals that $\Delta \omega_{diff}$ is proportional to the square root of the disturbance in the case of $\varepsilon \ll (\gamma_{t}-\gamma_{2})$.

**Figure 6:**
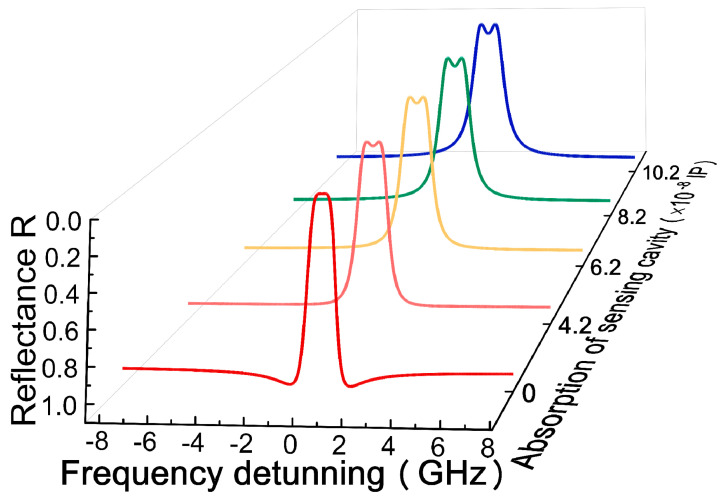
Splitting process of spectrum with the increase in absorption.

**Figure 7:**
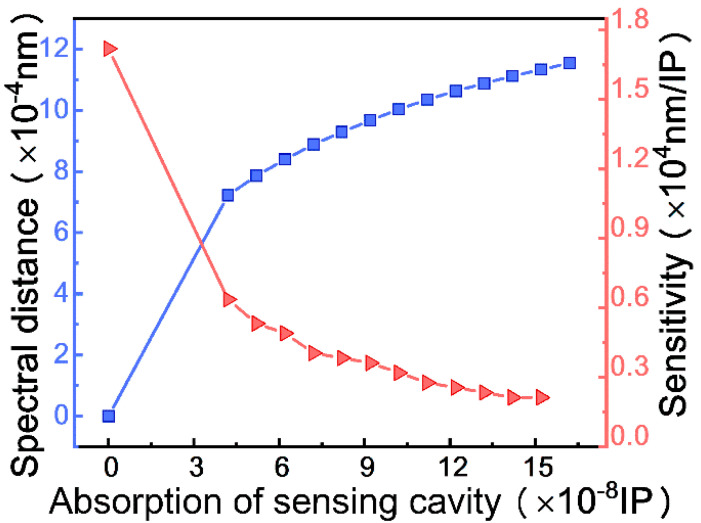
Graph of spectral distance (blue line) and sensitivity (red line) as a function of the imaginary part of RI of the cavity *S* representing the absorption.

Figure 6 shows the splitting process of the spectrum from a single dip to double dips near the EP when the absorption of the sensing cavity increases in response to the binding of the CEA on the surface of the sensing cavity. Consequently, strong absorption results in a large spectral distance. However, due to the square root feature near the EP, the sensitivity is higher for detecting the weak absorption, as shown in Figure 7. According to the simulation results above and the Rayleigh criterion, the corresponding theoretical minimum resolution for the imaginary part is 4.2 × 10^−8^ IP (the absorption coefficient is 3.4 × 10^−3^ cm^−1^), and the sensitivity reaches a factor of 17,120 nm/IP unit.

**Figure 8:**
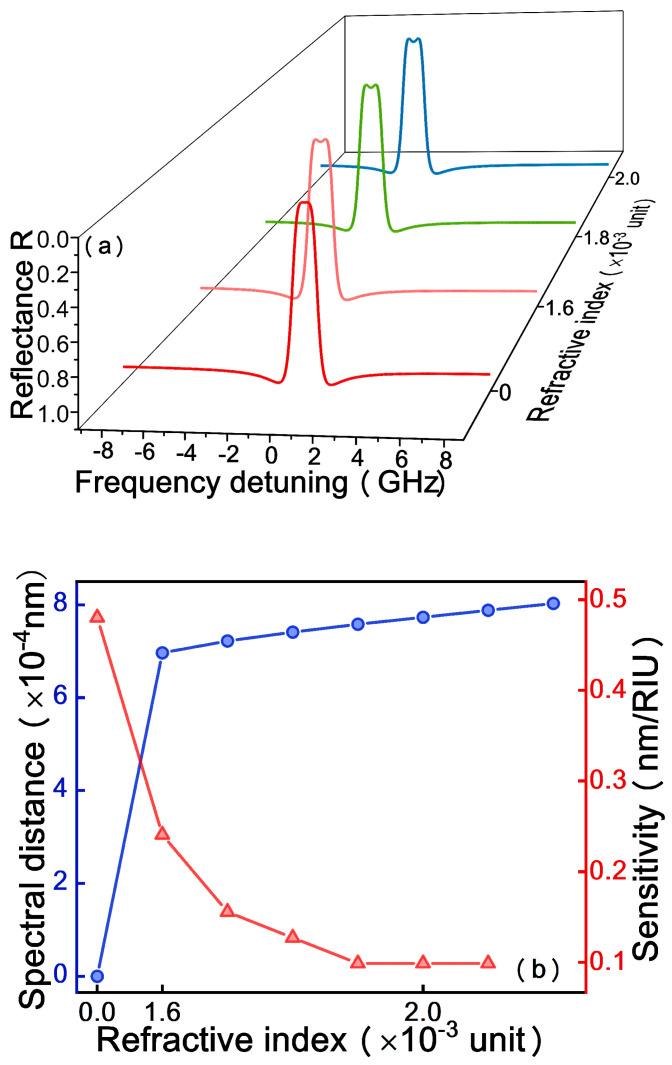
The influence of the refractive index(RI) of the coupling layer on the reflectance spectrum (**a**) and spectral distance (blue line) and sensitivity (red line) (**b**).

When the EBC is injected in the sample loading channel and CEA molecules are specifically bound on the surface of the sensing cavity, not only the absorption of the resonator *S* but also the RI of the coupling layer will vary. Figure 8a shows the simulation evolution of the output spectrum at EP with the change of RI of the coupling layer when biological analytes entering in the coupling layer, which is noted that the spectrum simultaneously has a redshift during the splitting process. Figure 8b shows the sensor performance of the variation in the RI of the coupling layer at the EP. As the RI of biological analytes increases, the single dip begins to split and the spectral distance increases gradually. However, the simulation results indicate that the sensitivity is 0.69 nm/refractive index unit (RIU), which is over 10^4^ times less than the sensitivity due to the absorption. Consequently, the RI of analytes has a minimal impact on the EP of the system.

To demonstrate the potential of the designed sensor, the sensing performance was evaluated. If the length of cavity *S* of the designed sensor is about 800 μm, the total absorption that can be obtained is 2.7 × 10^−4^. As the molar extinction coefficient of CEA is 1 × 10^5^ M^−1^ cm^−1^ [33] and the loss of CEA is 3.3 × 10^4^ cm^−1^, the thickness of the CEA can be detected to be about 8.25 × 10^−9^ cm. Assuming the cross-sectional area of the sensing cavity is 0.16 cm^2^ (the density of CEA is 1.35 × 10^3^ kg/m^3^), the theoretical minimum detection quantity of CEA is 1.78 ng. Setting that the EBC collection volume to be 2 mL, the CEA content is 4 ng for healthy people. Therefore, the theoretical detection limit of the designed sensor is lower than the typical amount of CEA. According to the simulation results, the sensor responds to a CEA content ranging from 1.78 to 14.52 ng. As the simulation only demonstrates an ideal result, the influence of quantum noise on the EP of the system should also be considered in an experiment [34]. However, the specific experimental protocol can overcome concerns about quantum noise by optimizing the signal-to-noise ratio [35].

**Figure 9:**
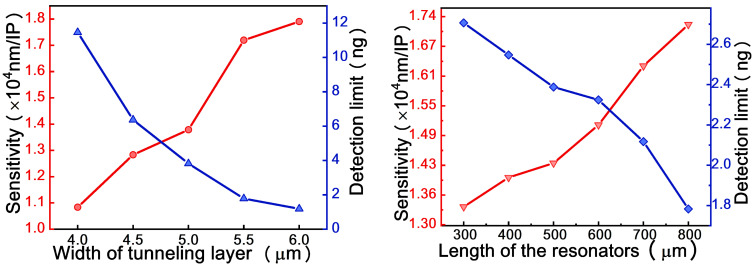
Effect of the width of tunneling layer (**left panel**) and the length of the resonators (**right panel**) on the detection limit (blue line) and the sensitivity (red line) near the exceptional point (EP).

Finally, some dimensions of the designed sensor, the width of the tunneling layer and cavity length, were investigated for the theoretical performance evaluation, as shown in Figure 9. When the width of the tunneling layer goes up, the sensitivity increases moderately, always keeping values above 1 × 10^4^ nm/IP. However, the detection limit of the sensor decreases markedly from 11.5 to 1.2 ng. In which refer to varying the length of the cavity, in a similar way as for the width, increasing the length leads to an increase of the sensitivity and a decrease on the detection limit. Nonetheless, the impact on the detection limit is smaller, changing from 2.7 to 1.8 ng. Consequently, the simulation results show that the detection limit and sensitivity of the sensor are improved under wider tunneling layer and longer resonator. As silicon wafer can be used as the ROTE resonator, on which the tunneling gap layer is formed by the spin coating process, the device can be fabricated with high fidelity. The manufacturing errors of these dimensions are far less than the values in the simulation, so the tolerances of the dimensions will not affect the performance of the designed sensor.

## 4. Conclusions

In this study, the EP was realized based on the coupled ROTE resonators system in simulation, near which the modes splitting response is proportional to the quasi-square root of the perturbation strength. By using this unique feature, a label-free biosensor was proposed to detect the concentration of CEA, and its sensing principle and performance were discussed theoretically. The simulation results demonstrate that the designed sensor has a theoretical detection limit of 4.2 × 10^−8^ IP, which corresponds to 1.78 ng of CEA. The sensitivity of the system can reach 17,120 nm/IP, which verifies that the sensor is able to detect the target compound in low concentrations. In addition to CEA detection, the designed sensor can find applications in the fields of environment protection, drinking water safety, and drug screening.

## Figures and Tables

**Table 1:** Parameters and materials of the coupled ROTE resonators.

Parameter	Material	Symbol	Value
RI of input prism	K9 glass	*n_in_*	1.5000−9.84 × 10^−8^*i*
RI of the first tunneling layer Width of the first tunneling layer	Polydimethylsiloxane (PDMS)	*n*_1_ *d*_1_	1.396–5 × 10^−6^*i* 5.5 μm
RI of the loss cavity Width of the loss cavity	Silicate glass	*n*_2_ *d*_2_	1.65−1.2 × 10^−8^*i* 800 μm
RI of coupling layer Width of coupling layer	Sample	*n*_3_ *d*_3_	1.3506−6.4538 × 10^−6^*i* 4.28 μm
RI of the sensing cavity Width of the sensing cavity	Silicate glass	*n*_4_ *d*_4_	1.65−1.6 × 10^−8^*i* 800 μm
RI of the second tunneling layer Width of the second tunneling layer	Polydimethylsiloxane (PDMS)	*n*_5_ *d*_5_	1.396–5 × 10^−6^*i* 5.5 μm
RI of output prism	K9 glass	*n_out_*	1.5000−9.84 × 10^−8^*i*
Incident angle		*θ*	68.84°

## Data Availability

Data sharing is not applicable to this article.
